# Patients’ Attitudes and Beliefs Toward Artificial Intelligence Use in Cancer Care: Cross-Sectional Survey Study

**DOI:** 10.2196/81346

**Published:** 2026-02-04

**Authors:** Marco Santos Teles, Karolina Bryl, Susan Chimonas, Atif Khan, Andrew S Epstein, Bobby Daly, Han Xiao, Jun J Mao

**Affiliations:** 1 Integrative Medicine Service, Memorial Sloan Kettering Cancer Center, 205 East 64th Street, New York, NY, 10022, United States, 1 646 608 8552; 2 Department of Epidemiology and Biostatistics, Memorial Sloan Kettering Cancer Center, New York, NY, United States; 3 Department of Radiation Oncology, Memorial Sloan Kettering Cancer Center, New York, NY, United States; 4 Gastrointestinal Oncology Service, Department of Medicine, Memorial Sloan Kettering Cancer Center, New York, NY, United States; 5 Thoracic Oncology Service, Department of Medicine, Memorial Sloan Kettering Cancer Center, New York, NY, United States; 6 Genitourinary Oncology Service, Department of Medicine, Memorial Sloan Kettering Cancer Center, New York, NY, United States

**Keywords:** patient perspectives, artificial intelligence, cancer care, digital health, patient attitudes

## Abstract

**Background:**

Artificial intelligence (AI) is being rapidly integrated into oncologic care, yet little is known about how patients perceive these applications. Understanding patient perceptions is critical to ensuring AI applications align with their needs and preferences.

**Objective:**

This study aimed to evaluate oncology patients’ attitudes and beliefs on the use of AI across clinical touchpoints in cancer care.

**Methods:**

We conducted a cross-sectional survey study with adult oncology patients from September to December 2024. The survey assessed patients’ comfort with AI use across 8 clinical touchpoints of cancer care (eg, screening, diagnosis, treatment) on a 5-point Likert scale (1=very uncomfortable to 5=very comfortable). Patients also rated their concerns about AI, including potential harms related to its use (eg, medical errors, privacy breaches), on a 3-point Likert scale (1=not concerned to 3=very concerned).

**Results:**

Of 383 patients approached, 330 (86.2% response rate) participated; 184 (55.9%) were male, 162 (49.4%) were aged 65 years or older, 35 (10.8%) were Black, 40 (12.1%) were Hispanic or Latino, and 233 (72.6%) were actively receiving cancer treatment. Patients were most comfortable with AI use in cancer screening (80.2%) and supportive care applications, including exercise (78.2%), diet (74.8%), and herbs/supplements (72.4%). Patients were least comfortable with AI use to assist with diagnosis (70.4%), symptom management (67.5%), treatment planning (64.8%), and prognosis (61.5%). Nonetheless, about half (49.7%) were at least somewhat concerned with the use of AI in cancer care, most commonly about the loss of human interaction and medical errors.

**Conclusions:**

Although the majority of oncology patients had a favorable view of AI in cancer care, nearly half had concerns about potential harms. Incorporating patient perspectives into AI development is essential for patient-centered and high-quality cancer care.

## Introduction

Artificial intelligence (AI) is transforming oncology, offering unprecedented capabilities to analyze vast amounts of cancer treatment data and improve care [1,2]. The integration of this technology into cancer care has centered on clinical touchpoints within the “cancer continuum,” such as diagnosis, prognosis, and treatment [2,3]. AI is already capable of performing tasks across these domains, including analyzing radiographic images, predicting clinical outcomes, and assisting in decision-making [4-6].

Despite these technological advances, little is known about how patients perceive the use of AI in cancer care. Prior research has focused on the views of the general population toward AI use in health care [7-9]. Although these studies suggest that patients are generally receptive to AI, data investigating views regarding its use in oncology are lacking. As the use of AI in oncology increases, understanding patient perceptions is essential to ensure their needs and preferences are incorporated into the implementation of AI applications. Failure to incorporate these perspectives may lead to AI tools that patients are unwilling to use, ultimately eroding trust in health care, jeopardizing the clinician-patient relationship, and reducing adherence to clinical recommendations.

To address this critical need for patient-centered implementation of AI, we conducted a cross-sectional survey with adult oncology patients at a large urban academic cancer center to evaluate their attitudes and beliefs on the use of AI in clinical touchpoints of cancer care.

## Methods

### Questionnaire Development

The survey was designed through a multistep process in collaboration with patients. First, a literature review informed question development on AI applications in cancer care, with some questions adapted from prior AI-in-medicine surveys [7,10,11]. A patient and family advisory council at our institution provided feedback on the survey draft. We then conducted cognitive interviews with 12 oncology patients in an outpatient clinic to assess language clarity and question relevance. The survey was then revised to incorporate patient-identified topics, including AI as a physician-supervised tool (vs standalone use), its clinical applications in oncology, and potential harms. Finally, we piloted the revised survey with 21 patients to test the survey recruitment feasibility in our clinical setting.

The finalized survey (Multimedia Appendix 1) consisted of 48 items. Participants rated their comfort with AI use across 8 clinical touchpoints of cancer care: screening, diagnosis, treatment, prognosis, symptom management, diet, herbs/supplements, and exercise. They also rated their concern with 5 potential AI use harms: privacy breaches, medical errors, loss of human interaction with doctors, difficulty understanding the technology to access care, and reinforcing discrimination in health care. Question formats included multiple choice, 5-point Likert scales (1=very uncomfortable to 5=very comfortable), and 3-point Likert scales (1=not concerned to 3=very concerned). Sociodemographic, clinical, and technology use data were collected. The survey was translated into Spanish by a certified interpreter.

### Survey Administration

This study used convenience clinical sampling. We distributed the survey to patients at affiliated outpatient clinics in urban academic, urban underserved, and suburban settings. Adult patients with a history of cancer were eligible to participate. Clinic staff screened patients and notified the research staff while patients waited for appointments or treatment. The research team introduced the study and provided patients with an iPad for the survey. The survey was distributed via Research Electronic Data Capture (REDCap) [12].

### Data Analysis

We followed American Association for Public Opinion Research reporting guidelines. Chi-square tests were used for subgroup analysis. Statistical Package for the Social Sciences (version 28; IBM Corp) was used for statistical analysis.

### Ethical Considerations

The study was reviewed by the institutional review board at Memorial Sloan Kettering Cancer Center and determined to be exempt from review under 45 Code of Federal Regulations (CFR) 46.104(d)(2)(i), which applies to research involving educational tests, survey procedures, interview procedures, or observation of public behavior when participant identities cannot readily be ascertained. The exemption determination was issued on September 5, 2024 (protocol number X24-033). Given the anonymous nature of the survey, written informed consent was not required. Participants were presented with an electronic consent statement at the start of the survey, and consent was obtained by participants selecting “I agree” before proceeding. No compensation was provided to participants.

## Results

Between September and December 2024, 383 patients were approached, of whom 330 participated (86% response rate). Of these 330 participants, 184 (55.9%) were male, 162 (49.4%) were aged 65 years or older, 35 (10.8%) were Black, and 40 (12.1%) were Hispanic or Latino (Table 1). The most commonly reported cancers were prostate (114/330, 34.5%) and breast (87/330, 26.4%) cancer, and the majority of patients were actively receiving treatment (233/321, 72.6%).

**Table 1.** Participants’ demographics and baseline characteristics (N=330).

Characteristic		Participants, n^a^ (%)
Age (years)		
	18‐49	58 (17.7)
	50‐64	108 (32.9)
	≥65	162 (49.4)
Gender		
	Male	184 (55.9)
	Female	141 (42.9)
	Other	4 (1.2)
Race		
	White	234 (72.4)
	Black/African American	35 (10.8)
	Asian	25 (7.7)
	Other	29 (9)
Ethnicity		
	Hispanic	40 (12.4)
	Non-Hispanic	283 (87.6)
Education		
	High school degree or less	73 (22.6)
	College degree or more	250 (77.4)
Cancer type^b^		
	Prostate	114 (34.5)
	Breast	87 (26.4)
	Gastrointestinal	52 (15.8)
	Genitourinary	33 (10)
	Lung	22 (6.7)
	Other	66 (20)
Treatment status		
	Receiving treatment	233 (72.6)
	Not receiving treatment	88 (27.4)
Treatment modality^b^		
	Surgery	158 (47.9)
	Radiation	180 (54.5)
	Chemotherapy	209 (63.3)
	Immunotherapy	64 (19.4)
	Other	60 (18.2)
Self-rated general health		
	Excellent to good	272 (83.7)
	Fair to poor	53 (16.3)

a Counts may not add up to 330 due to missing data (<5% missing data for each question).

b This is a “select all that apply” question. Patients could report multiple cancers/treatment modalities.

**Figure 1.**
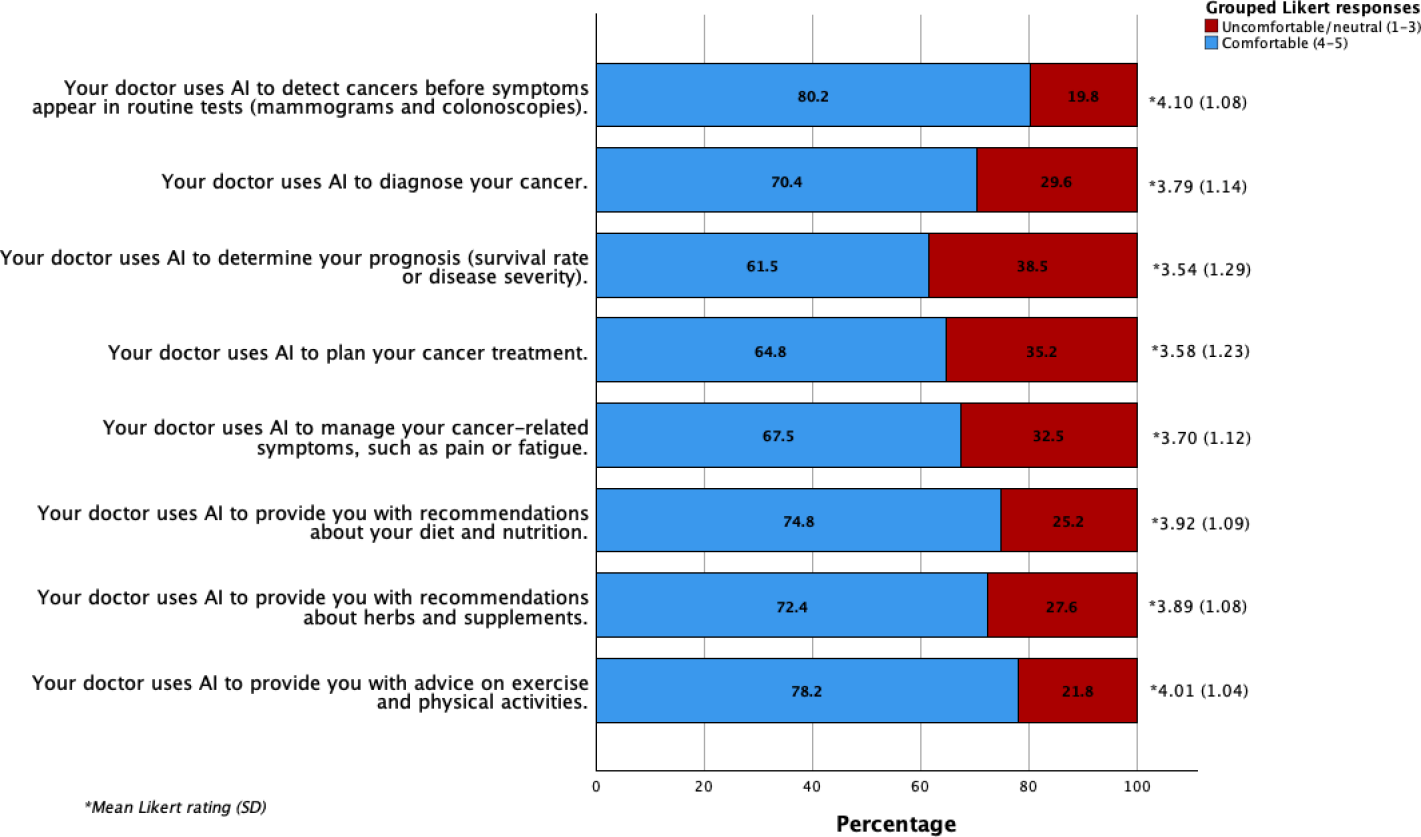
Patients’ comfort level with AI use in different clinical touchpoints of cancer care. AI: artificial intelligence.

Most participants (300/330, 91%) reported knowing “a little” or “quite a lot” about AI, and 60 of 312 participants (19.2%) used web-based AI applications (eg, ChatGPT) at least once per week. The majority (264/328, 80.5%) believed that AI would make cancer care somewhat or much better over the next 5 years. Patients were most comfortable with AI use in cancer screening (264/329, 80.2%) and supportive care applications including recommendations for exercise (254/325, 78.2%), diet (247/330, 74.8%), and herbs/supplements (236/326, 72.4%). Patients were least comfortable with AI use to assist with diagnosis (231/328, 70.4%), symptom management (222/329, 67.5%), treatment planning (210/324, 64.8%), and prognosis (201/327, 61.5%) (Figure 1).

Nevertheless, 49.7% (164/330) were somewhat concerned with the use of AI in their cancer care. The most common concerns were loss of human interaction with doctors (162/330, 49.1%) and medical errors (158/330, 47.9%), followed by privacy breaches (141/330, 42.7%), difficulty understanding the technology (132/330, 40%), and reinforcing health care disparities (112/330, 33.9%).

Compared to patients who reported limited AI use, those who used it weekly were more likely to believe AI would improve cancer care (57/60, 95% vs 194/25, 77.3%; *P*=.006) and were less concerned about its use (18/60, 30% vs 135/252, 53.6%; *P*=.001). Compared to patients with less than a college degree, those with a college degree or higher were more likely to believe that AI would improve cancer care (53/73, 72.6% vs 207/248, 83.5%; *P*=.04), but not more likely to be concerned about its use (36/73, 49.3% vs 124/250, 49.6%; *P*=.97). Treatment status was not associated with differences in perceptions. Patients on active treatment were no more likely than those not on active treatment to believe that AI would positively impact cancer care (191/233, 82% vs 65/86, 75.6%; *P*=.20) or to express concerns about its use (114/233, 48.9% vs 44/88, 50%; *P*=.86).

## Discussion

### Principal Findings

AI holds significant potential to improve oncologic care, though successful integration of this technology must incorporate patients’ perspectives, as they are the ultimate decision-makers in their care. This study highlights opportunities and challenges to optimal AI implementation in cancer care.

Although most oncology patients were receptive to AI applications, nearly half expressed concerns about potential harms, particularly loss of human interactions and medical errors. These findings align with prior studies in the general population, reflecting “cautious optimism”—patients are open to AI but emphasize the need for oversight to mitigate risks [7-9]. The most common concerns relate to previously raised ethical implications: (1) AI jeopardizing clinician-patient relationships leading to depersonalized care, and (2) overreliance on clinical support tools causing inaccurate diagnoses and recommendations [13]. These concerns echo efforts in the regulatory landscape, including the US Food and Drug Administration’s evolving oversight of AI-based medical software and the EU AI Act, which designates most medical AI systems as “high-risk,” requiring additional oversight measures [14]. Policymakers and developers must engage patients during AI implementation to ensure safeguards are in place to address these concerns.

Our findings demonstrate a higher proportion of patients that were comfortable with AI than previously reported. For example, a 2019 survey of the general population found 55.4% believed AI would improve health care; in our study, 80.5% of oncology patients had this expectation [7]. Similarly, a 2022 survey found 39% of US adults were comfortable with their health care providers relying on AI for their medical care [15], while in our study, at least 60% of patients were comfortable with their doctors using AI for various clinical tasks in cancer care. These discrepancies may reflect increasing familiarity with AI in the last few years. Our findings suggest exposure to this technology may reduce patient concerns. Additionally, we observed that patients with a college degree or higher were more likely to have positive views toward the future of AI in cancer care. This is in line with another recent study that showed patients with higher education levels expressed less discomfort with AI and fewer concerns about AI technologies in clinical settings [16]. Future interventions should explore whether targeted exposures for older or less educated patients could narrow the digital divide in clinical settings.

### Limitations

A limitation of this study is reliance on convenience sampling of patients at a single center in the Northeastern United States, making the sample less representative of the US cancer patient population. Furthermore, while we had a robust response rate, in-person responses may have introduced social desirability bias, as patients may have expressed more favorable views due to perceived health care expectations. The education level of our participants (77.4% with a college degree or more) was higher than the national average [17] and potentially associated with more positive views toward AI in cancer care, which may impact the generalizability of our findings.

### Conclusion

Although most oncology patients had positive views of AI applications in cancer care, almost 50% had concerns, especially about loss of interaction with clinicians and potential medical errors. To optimize patient-centered care, policymakers and developers must incorporate patient input during AI implementation and address these concerns. Future research should seek to identify strategies to foster collaboration between developers and patients. Importantly, patient perceptions of AI may shift over time as public discourse and clinical exposure to AI tools evolve; therefore, a longitudinal study may better assess how these views change over time as AI becomes more integrated into oncology.

## Supplementary material

10.2196/81346 Multimedia Appendix 1 Survey on artificial intelligence in cancer care delivery.

## Abbreviations

AI: artificial intelligence
REDCap: Research Electronic Data Capture
