# Supplementary material for: Patients’ Attitudes and Beliefs Toward Artificial Intelligence Use in Cancer Care: Cross-Sectional Survey Study
Source: JMIR Cancer. 2026 Feb 4;12:e81346. doi: 10.2196/81346 (PMC12872120; doi:10.2196/81346)
Supplement: Multimedia Appendix 1 [file cancer-v12-e81346-s001.doc]

*Page 1*

**Artificial Intelligence in Cancer Care Delivery**

This survey seeks to understand your thoughts on how artificial intelligence (AI) is used in cancer care. AI refers to technology that can imitate human thinking, like learning, reasoning, and problem-solving. Your feedback is incredibly valuable in helping us improve the experience of cancer care for patients like you who may encounter AI.

Your participation in this study is voluntary and anonymous. The survey should take approximately 5 minutes to complete. Please indicate whether you agree to take part in this study. Thank you for your participation!

Please click on one of the buttons to indicate whether you agree to take part in this study.

I agree


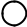


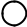
 I do not agree

**How often do you use each of the following electronic devices?**

| Every day | 3 to 4 times a | 1 to 2 times a | 1 to 2 times a | Less than once a |
| --- | --- | --- | --- | --- |
|  | week | week | month | month, or never |

Computer (laptop or desktop)


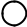

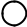

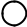

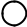

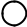


Tablet (Ipad, Android tablet)


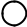

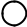

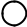

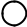

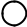


Smartphone (Iphone or Android)


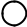

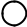

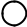

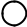

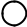


Home assistant ("Alexa, " "Siri,"


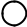

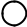

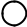

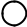

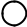


"Hey Google...")

Web-based AI applications


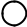

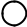

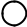

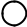

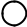


(ChatGpt, Microsoft Copilot)

**How Artificial Intelligence (AI) can be used in cancer care**

**AI has been tested in medicine to assist in tasks normally done by your doctor alone, such as making treatment recommendations and diagnoses. With this information in mind, please answer the following questions.**

How much do you know about what AI is?


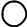
 I never heard of it


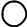
 I know a little bit


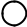
 I know quite a lot

Over the next 5 years, do you think AI will make cancer care in the United States _______


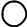
 Much worse


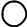
 Somewhat worse


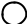
 No change ( I don't know what AI is)


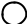
 Somewhat better


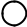
 Much better

**How comfortable would you feel if your doctor used AI for the following aspects of your cancer care?**

| Very | Somewhat | Neutral | Somewhat | Very comfortable |
| --- | --- | --- | --- | --- |
| uncomfortable | uncomfortable |  | comfortable |  |

Your doctor uses AI to detect


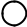

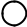

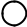

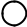

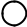


cancers before symptoms

appear in routine medical tests,

such as mammograms and

colonoscopies.

Your doctor uses AI to diagnose


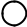

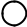

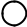

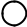

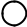


your cancer.

Your doctor uses AI to determine


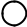

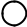

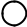

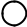

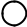


your prognosis (survival rate or

disease severity).

Your doctor uses AI to plan your


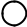

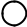

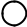

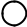

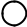


cancer treatment.

Your doctor uses AI to manage


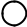

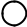

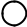

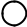

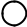


your cancer-related symptoms,

such as pain or fatigue.

Your doctor uses AI to provide


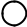

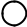

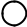

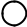

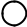


you with recommendations

about your diet and nutrition.

Your doctor uses AI to provide


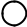

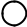

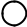

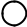

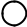


you with recommendations

about herbs and supplements.

Your doctor uses AI to provide


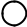

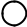

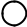

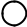

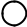


you with advice on exercise and

physical activities.

How concerned are you about the use of AI in your cancer care?


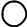
 Not concerned at all


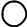
 Somewhat concerned


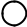
 Very concerned

How concerned are you with each of these issues if AI were used in your cancer care? (**Branching logic:** only if participant is concerned with AI in cancer care)

Not concerned at all Somewhat concerned Very concerned

Privacy breaches


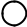

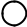

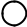


Medical errors


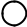

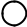

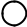


Loss of human interactions with


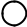

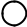

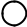


doctors

Difficulty understanding the


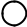

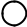

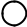


technology to access care

Reinforcing discrimination and


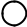

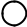

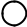


disparities in healthcare

Please feel free to share any more thoughts or opinions you have about AI in cancer care here:

**Demographic Information:**

How old are you?


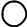
 18-29


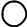
 30-49


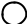
 50-64


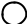
 65-79


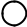
 80 or older

What is your gender?


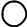
 Male


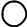
 Female

Transgender

Nonbinary

Other

Prefer not to say

Please specify

__________________________________

Which of the following best describes your racial identity? Select all that apply.

American Indian or Alaska Native

Asian

Black or African-American

Native Hawaiian or Other Pacific Islander

White or Caucasian

Another race not listed here

Please specify:

__________________________________

Are you Hispanic or Latino?

Yes

No

What is the highest level of education you have completed?

Some high school or less

Graduated from high school or equivalent

Graduated from college

Completed graduate or professional degree

**Cancer History:**

Which type(s) of cancer do/did you have? Select all that apply.

Breast cancer

Colon cancer

Kidney cancer

Leukemia or lymphoma

Lung cancer

Melanoma

Mouth/tongue/lip/throat cancer

Ovarian cancer

Pancreatic cancer

Prostate cancer

Skin cancer - Not melanoma

Stomach cancer

Thyroid cancer

Other

Please specify:

__________________________________

At what age were you diagnosed with your most recent cancer?

__________________________________

Are you currently receiving treatment for your cancer?

Yes

No, I have completed treatment

No, I have not started treatment yet

Treatment was not necessary

What types of treatments have you received?

Surgery

Radiation therapy

Chemotherapy

Chemotherapy pills

Immunotherapy

Other

Please specify:

__________________________________

Would you say that in general your health is ________

Excellent

Very good

Good

Fair

Poor
